# Simulating real world functioning in schizophrenia using a naturalistic city environment and single-trial, goal-directed navigation

**DOI:** 10.3389/fnbeh.2013.00180

**Published:** 2013-11-26

**Authors:** John A. Zawadzki, Todd A. Girard, George Foussias, Alicia Rodrigues, Ishraq Siddiqui, Jason P. Lerch, Cheryl Grady, Gary Remington, Albert H. C. Wong

**Affiliations:** ^1^Institute of Medical Science, University of TorontoON, Canada; ^2^Centre for Addiction and Mental Health, Campbell Family Mental Health Research InstituteToronto, ON, Canada; ^3^Department of Psychology, Ryerson UniversityToronto, ON, Canada; ^4^Department of Psychiatry, University of TorontoON, Canada; ^5^Collaborative Program in Neuroscience, University of TorontoON, Canada; ^6^Department of Medical Biophysics, University of TorontoON, Canada; ^7^Program in Neuroscience and Mental Health, The Hospital for Sick ChildrenToronto, ON, Canada; ^8^Departments of Psychology and Psychiatry, University of TorontoON, Canada; ^9^Rotman Research Institute at BaycrestToronto, ON, Canada

**Keywords:** schizophrenia, cognition, psychosocial functioning, virtual reality, navigation

## Abstract

**Objective:** To develop a virtual reality platform that would serve as a functionally meaningful measure of cognition in schizophrenia and that would also complement standard batteries of cognitive tests during clinical trials for cognitive treatments in schizophrenia, be amenable to human neuroimaging research, yet lend itself to neurobiological comparison with rodent analogs.

**Method:** Thirty-three patients with schizophrenia and 33 healthy controls matched for age, sex, video gaming experience, and education completed eight rapid, single-trial virtual navigation tasks within a naturalistic virtual city. Four trials tested their ability to find different targets seen during the passive viewing of a closed path that led them around different city blocks. Four subsequent trials tested their ability to return to four different starting points after viewing a path that took them several blocks away from the starting position.

**Results:** Individuals with schizophrenia had difficulties in way-finding, measured as distance travelled to find targets previously encountered within the virtual city. They were also more likely not to notice the target during passive viewing, less likely to find novel shortcuts to targets, and more likely to become lost and fail completely in finding the target. Total travel distances across all eight trials strongly correlated (negatively) with neurocognitive measures and, for 49 participants who completed the Quality of Life Scale, psychosocial functioning.

**Conclusion:** Single-trial, goal-directed navigation in a naturalistic virtual environment is a functionally meaningful measure of cognitive functioning in schizophrenia.

## Introduction

Schizophrenia is a chronic mental illness presenting with psychotic symptoms of delusions and hallucinations against a background of neurocognitive impairment, poor motivation, and poor psychosocial functioning (Heinrichs and Zakzanis, 1998; Wong et al., 2003). Cognitive deficits are a significant predictor of functional outcome (Green, 1996; Harvey et al., 1998; Green et al., 2000, 2004), and are current targets for psychopharmacological treatment (Hyman and Fenton, 2003).

A number of neuropsychological tests assess cognitive domains that are particularly impaired in schizophrenia (Nuechterlein et al., 2004). These have been incorporated into a standard battery of cognitive tests for use in clinical trials by the Measurement and Treatment Research to Improve Cognition in Schizophrenia (MATRICS) initiative (Nuechterlein et al., 2008). In order to provide tools for clinical trial evaluation, the Cognitive Neuroscience Treatment Research to Improve Cognition in Schizophrenia (CNTRICS) initiative identified additional tests from cognitive neuroscience literature that are sensitive to schizophrenia (Barch et al., 2009). However, the US Food and Drug Administration stipulates a requirement for a functionally meaningful measure of overall outcome in addition to an accepted battery of cognitive tests for clinical trials for cognitive treatments in schizophrenia (Green et al., 2011).

Goal-directed navigation offers a powerful paradigm for studying neural system interactions during complex human behaviors (Spiers and Maguire, 2006). Rodent models have detailed the neurobiology of various cognitive processes including perception, motivation, planning, and decision making at molecular, cellular, and systems level analysis (Burgess, 2008; Moser et al., 2008; Penner and Mizumori, 2012). The neurotransmitter dopamine, an important target in schizophrenia research (Laruelle and Abi-Dargham, 1999), plays a dominant role in the modulation of the neuroanatomical structures and networks engaged in rodent navigation (Penner and Mizumori, 2012).

Several core brain regions involved in successful goal-directed navigation in humans (Aguirre et al., 1996; Wolbers et al., 2004; Wolbers and Hegarty, 2010) including the hippocampus, prefrontal cortex and striatum (Maguire et al., 1998; Astur et al., 2002; Driscoll et al., 2003; Ekstrom et al., 2003; Hartley et al., 2003; Iaria et al., 2003; Bohbot et al., 2004; Voermans et al., 2004; Spiers and Maguire, 2006; Doeller et al., 2008; Brown et al., 2012), are also strongly implicated in the pathophysiology of schizophrenia (Weinberger et al., 1986; Goldberg et al., 1987; Weinberger, 1987; Bogerts et al., 1990; Csernansky et al., 1999; Laruelle and Abi-Dargham, 1999). Optimum navigation is associated with the ability to flexibly switch between hippocampal and striatal networks (Hartley et al., 2003; Iaria et al., 2003; Etchamendy and Bohbot, 2007) and to integrate wayfinding networks with prefrontal cortex working memory (Wolbers et al., 2007; Hanlon et al., 2012) and executive functions (Maguire et al., 1998; Hartley et al., 2003). The virtual reality (VR) navigation paradigms used for schizophrenia research, by focusing on specific neural structures or cognitive domains, have tended to be based on rodent models or necessarily circumscribed environments within a paradigm of trial and error learning or extensive exploratory activity prior to testing to ensure familiarity with landmark locations (Astur et al., 2004; Hanlon et al., 2006, 2012; Weniger and Irle, 2008; Folley et al., 2010; Spieker et al., 2012).

We sought to extend this work by designing a more realistic human VR task that would assess goal-directed navigation in a naturalistic city environment. In addition, rather than using a multiple-trial incremental learning paradigm, we developed a single-trial paradigm more analogous to daily events such as going to a shopping mall and trying to find the shop you spotted on your way to the drug store or trying to find your way back to your parked car. Here we report on the results of the behavioral testing of performance by patients with schizophrenia using a single-trial navigation paradigm in a naturalistic virtual city.

## Materials and methods

### Participants

All experiments were conducted with the approval of our institutional research ethics board. Informed consent was obtained from all participants. Diagnosis was based on the Mini International Neuropsychiatric Inventory (MINI) (Sheehan et al., 1998) which enables diagnosis based on DSM-IV. Thirty-three outpatients diagnosed with schizophrenia (*n* = 22) or schizoaffective disorder (*n* = 11) and on stable doses of antipsychotics for the preceding 4 weeks took part in this study. All patients were chronically ill with durations of illness ranging from 2 to 39 years (*M* = 15, median = 11.5). Thirty-three healthy controls were individually matched with patients for age, sex (total 42 male, 24 female), and experience with first person action computer games. As a group, patients, and controls were also matched in education (Table 1).

**Table 1:** **Patients with schizophrenia were individually matched with healthy controls for age (within 4 years), sex, and video gaming experience (within 1 level of difference)**.

	**Age**		**Gaming experience**		**Education**		**WAIS-III pro-rated FSIQ**	
	**SZ**	**HC**	**SZ**	**HC**	**SZ**	**HC**	**SZ**	**HC**
Mean	40	39	0.8	0.7	4.1	4.2	107	116
Median	43	43	0	1	4	4	108	116
Standard deviation	10.9	11.3	1.3	0.9	0.9	1.0	17.8	14.0
Range	21–54	21–55	0–4	0–4	2–5	2–5	78–137	91–145

As a group they were also matched for education. HC, healthy controls; SZ, patients with schizophrenia; FSIQ, pro-rated full scale IQ. Gaming experience: 0 = never, 1 = a few times per year, 2 = a few times per month, 3 = a few times per week; 4 = daily; Education: 1 = less than high school, 2 = some high school, 3 = completed high school, 4 = some post-secondary, 5 = completed post-secondary.

Patients were recruited from the Center for Addiction and Mental Health, Toronto, Canada. Healthy controls were recruited by advertisements within the local community. Exclusion criteria for healthy controls were—no personal or family history of psychiatric dysfunction; for patients—no other DSM-IV Axis I disorder. Inclusion/exclusion criteria for both groups were: (1) 18–55 years of age; (2) no history of substance abuse in the past 3 months; (3) no history of neurological disease or loss of consciousness longer than 15 min; (4) a Wechsler Adult Intelligence Scale-III (WAIS-III) (Wechsler, 1997) prorated full scale IQ score greater than 75; and, (5) fluency in English.

### Clinical and neuropsychological assessment

Current positive and negative symptoms were assessed by using the Scales for the Assessment of Positive Symptoms (SAPS) and Negative Symptoms (SANS) (Andreasen, 1983, 1984). General intellectual ability was assessed using four subscales (vocabulary, similarities, block design, and matrix reasoning) of the WAIS-III (Wechsler, 1997), prorated to obtain estimated full scale IQ scores (Schrimsher et al., 2008). Participants also completed the Repeatable Battery for the Assessment of Neuropsychological Status (RBANS; Randolph, 1998). Education level was classified along five categories: “less-than-high-school,” “some high school,” “completed high school,” “some post-secondary,” and, “completed-post- secondary.” Forty-nine of the participants in this study (twenty-five with schizophrenia and twenty-four healthy controls) participated in a concurrent study that used the Quality of Life Scale (Heinrichs et al., 1984) to evaluate current psychosocial status. Those results were incorporated into this study. Clinical evaluations were completed during one session, typically 1–2 weeks prior to a single session of cognitive, and navigation trials.

### Virtual reality environment

**Figure 1:**
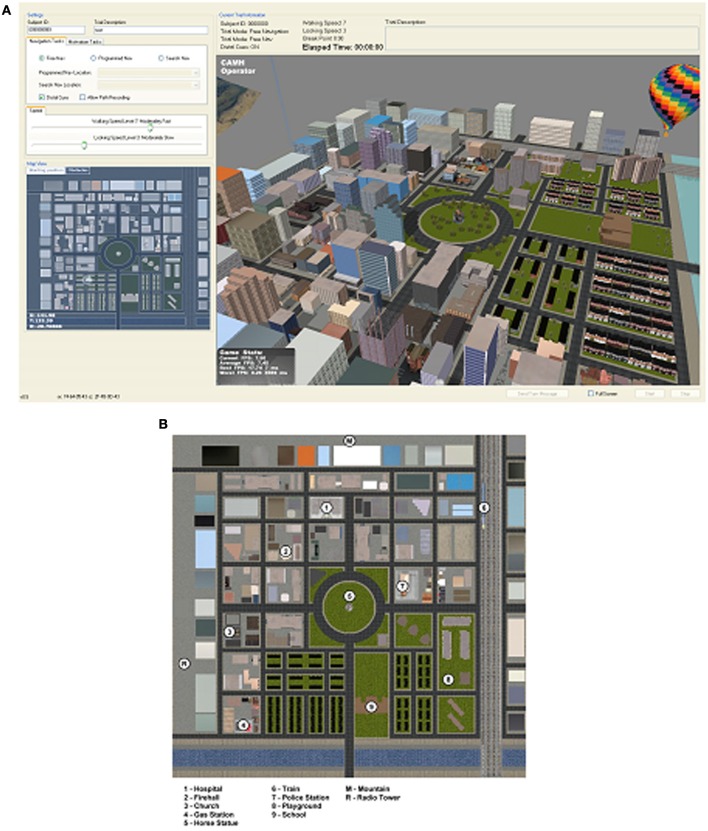
**Virtual city. (A)** Screen shot of the VR software operator showing an aerial map view of the virtual city and a 3-D isometric projection of the 6 × 6 block city. **(B)** Detailed aerial view showing the location of a few landmarks. These views were not seen by the participants.

**Figure 2:**
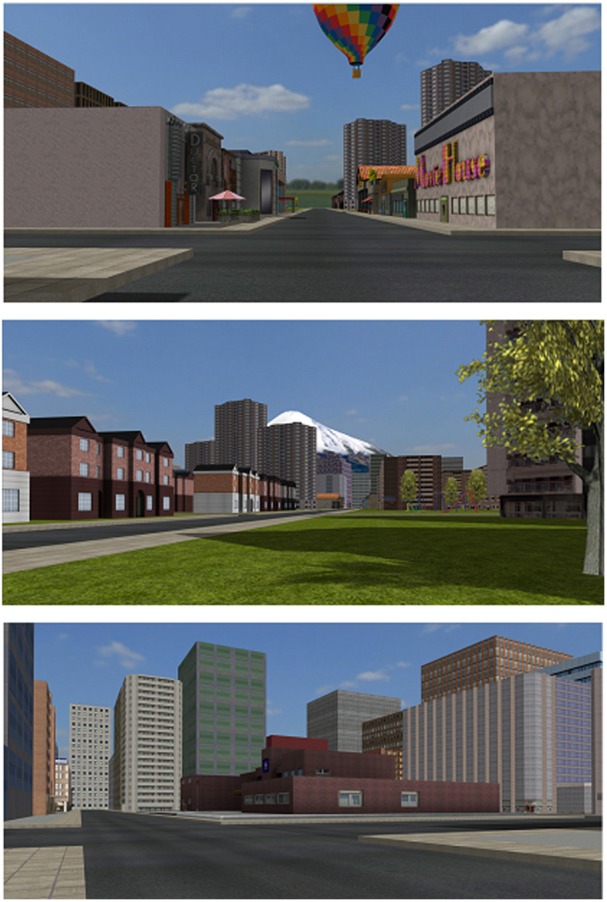
**Virtual city**. Examples of subject views of the environment are shown.

The VR environment consisted of a 6 x 6 block cityscape with a central park and over 80 residential, commercial, institutional, and office buildings. Embedded within the city were various “targets” such as a playground and hospital (Figures 1, 2). The environment included distal cues to aid in orienting such as a mountain range along one boundary, a hot air balloon, and a tall radio tower. Participants used a video game controller with simple forward/reverse and left/right levers to navigate. A 5 min practice session of free navigation before the beginning of trials, restricted to an area of the virtual city not used during subsequent trials, ensured familiarity with controller operations.

### Navigation trials

**Figure 3:**
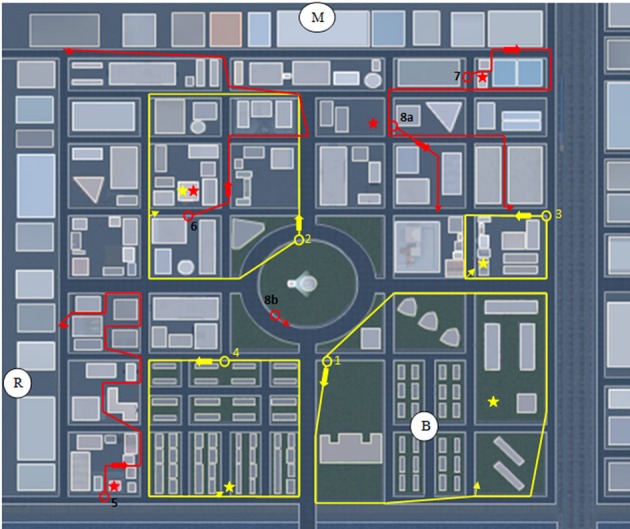
**Prerecorded paths viewed by participants during the passive viewing of each trial**. Shown is the aerial view of each path. During eye-level passive viewing the paths shown in yellow (four closed loop paths numbered according to order of presentation) and red (four return paths, similarly ordered) were followed by the participant starting at the open circle. For the closed loop trials the star represents the target and the small arrow pointing to the target indicates the position along the path where path movement temporarily halted and attention was directed toward the target for 2–3 s before proceeding to the end of the path. For the return path trials the star represents the target focused on at the start of the trial and the small arrow at the end of the path the direction of view at the end of the prerecorded path. At the end of the passive viewing portion of each trial the participant was asked to locate the target using the shortest route they could think of, beginning where the passive viewing portion ended. 8a represents the passive viewing portion of trial 8, ending at a position affording a view of the overhead balloon (B) and radio tower (R); 8b represents the starting position, facing the overhead balloon, for the subsequent attempt, during trial 8, by the participant to return to the starting position at 8a. M, mountain range.

A total of eight different navigation trials were carried out, each within different areas of the VR city. Each trial commenced with passive viewing of a pre-recorded path taken along several blocks of the virtual city, followed by a single attempt by the participant to locate a target shown during the passive viewing portion of the trial. The first four trials assessed participant ability to find a target seen during the passive viewing of a closed path that led them in a loop around one or more city blocks (“closed-loop” trials, Figure 3). The second four trials assessed participant ability to return to a starting point after viewing a path that took them a few blocks away from the starting position (“return-path” trials, Figure 3). All trials were presented in the same fixed random order to all participants.

For all trials, participants were instructed to take the shortest route possible to find the targets. Participants were asked to confirm that they had noticed the target immediately after passive viewing in each of the trials. Targets were typically focused on for 2–3 s during path viewing. Those who failed to see the target were allowed to view the pre-recorded path a second time. For those who failed to find the target, distances travelled were measured as total distances travelled until the participant admitted being lost and gave up further searching.

Each of the navigation trials took approximately 2–5 min to complete. Completion of all 8 trials ranged from a low of 15 min to a high of 60 min with an overall average of 30 min for patients and 22 min for controls.

### Hardware and software

The VR environment was rendered and presented on the testing computer to participants using an advanced graphics workstation and a 30′ widescreen LCD display. A separate computer, connected via a local area network to the testing computer, controlled task settings for each trial. The scene assets for the 3D virtual city were created in Maya and Adobe Photoshop. The simulation was rendered using Ogre, an open source 3D graphics engine. Data on distance travelled, time on task, and path directions travelled were stored automatically throughout the trials, and a video recording of the path followed by participants was made during each trial.

### Statistical analyses

Success at finding targets or returning to original starting positions was measured as the distance travelled to find the target or return to origin. Non-parametric statistics were used because all trials showed non-normal distributions in total distances travelled, with Shapiro-Wilk statistics typically significant at *p* < 0.01. The two matched samples were compared using the Wilcoxon Signed-ranks test (SRT) which yields a Z-statistic. Corresponding effect sizes were calculated as the SRT *Z*-value divided by the square root of sample size. Correlation analysis (Spearman's rho) was used to assess the relationships between distances travelled across all eight trials and scores on clinical (SAPS/SANS), cognitive (WAIS-III and RBANS), and psychosocial functioning (Quality of Life) measures. A logistic regression was undertaken to determine the role played by VR performance (distance travelled) in predicting group membership as compared to cognitive testing (RBANS total score, prorated WAIS-III full scale IQ), and psychosocial functioning (total score on the Quality of Life Scale). Pearson's r was used to assess relationships between normally distributed cognitive and clinical symptom scores. Correlations between task performance (distance travelled) and cognitive tests (RBANS, pro-rated WAIS-III full scale IQ) as well as the Quality of Life Scale scores were conducted on all subjects. Regression analysis of task performance with cognitive and psychosocial tests similarly represents all subjects. Correlations with clinical tests (SAPS/SANS) were conducted only on patients. Results were evaluated at an alpha-level of 0.05. SPSS v13.0 was used for the statistical analysis.

## Results

**Figure 4:**
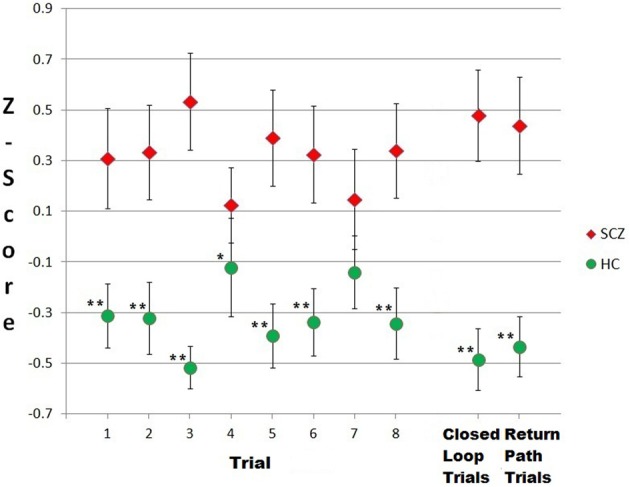
**Individuals with schizophrenia travelled further to find targets in a virtual city than healthy controls**. Shown are the standardized distances (z-scores) travelled across each of eight trials. Group means and standard errors are illustrated. Trials 1–4 = closed loop trials; trials 5–8 = return path trials. Asterisks represent between-group comparisons; ^*^p ≤ 0.05; ^**^p < 0.01.

**Figure 5:**
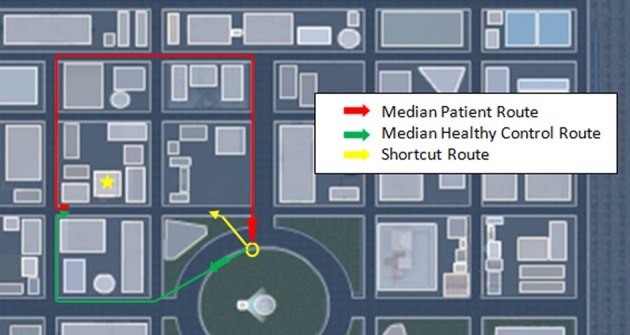
**Individuals with schizophrenia used longer routes to find targets than healthy controls**. Shown are the median distances travelled and their respective routes by patients and healthy controls for closed loop trial #2. The target, identified by the yellow star, is a fire hall. The yellow circle marks the trial start position. Participants unable to recognize a shortcut route typically attempted to retrace the forward path seen during passive viewing or attempted a reverse path to the target. Errors typically occurred when individuals failed to turn at the proper intersection.

In order to compare performances across trials, distances travelled were standardized by conversion to z-scores (Figure 4). A Friedman test of the four closed-loop trials indicated that there were no significant differences in difficulty between the trials for either schizophrenia [χ^2^_(3)_ = 0.82, *p* = 0.85] or control subjects [χ^2^_(3)_ = 3.22, *p* = 0.37]. Similarly, a Friedman test of the four return-path trials showed no significant differences in difficulty for either schizophrenia [χ^2^_(3)_ = 2.56, *p* = 0.47] or healthy participants [χ^2^_(3)_ = 7.01, *p* = 0.07]. Distances across each of the four trials were therefore added together for a composite distance travelled score for the closed-loop and return-path trials. Across the four closed-loop trials, patients travelled significantly further (median = 9564) than their matched healthy controls (median = 5467), *Z* = −4.05, *p* < 0.001, *r* = 0.50 (Table 2). Across the four return-path trials the patient group again travelled significantly further (median = 19,214) than controls (median = 12,579), *Z* = −3.58, *p* < 0.001, *r* = 0.44 (Table 2). Figure 5 illustrates, using one of the closed loop trials, differences between the two groups' path trajectories based on the representative median for each group.

**Table 2:** **A comparison of mean differences between patient and control groups illustrates the ability of goal-directed navigation to obtain results similar to that of standard neurocognitive measures**.

	**SCZ**		**HC**		**Difference in group means(a)**
	**Mean**	***SD***	**Mean**	***SD***	
**NAVIGATION TRIALS**					
1–4 total	10,328	4611	6029	3128	− 0.96
5–8 total	20,856	8606	14,115	5350	− 0.86
1–8 total	31,185	11,045	20,143	7065	− 1.03
**RBANS**					
Immediate memory	92	20	105	14	− 0.71
Visuospatial/Constructional	86	17	104	15	− 0.95
Language	94	13	101	14	− 0.53
Attention	89	18	104	14	− 0.88
Delayed memory	88	17	101	8	− 0.91
Total	86	15	105	14	− 1.08
**WAIS-III**					
Pro-rated verbal IQ	109	21	113	14	− 0.27
Pro-rated performance IQ	105	18	116	16	− 0.63
Pro-rated full scale IQ	107	18	116	14	− 0.54

Distances are expressed in virtual units wherein 500 units = approximately 1 virtual block; N = 66; (a) = scores were standardized prior to group comparisons; HC, healthy controls; SCZ, individuals with schizophrenia. For consistency, all variables were scored such that (−) differences represent a schizophrenia-related deficit.

The time parameter had similar results. Across the four closed-loop trials, patients took significantly longer in their attempts to find their targets (median = 8.3 min., range = 2.6–20.4 min.) than their matched healthy controls (median = 4.4 min., range = 1.9–14.5 min.), *Z* = −3.92, *p* < 0.001, *r* = 0.48. Across the four return-path trials the patient group again took significantly longer (median = 13.2 min, range = 5.0–38.0 min.) than controls (median = 8.5 min., range = 4.0–22.0 min.), *Z* = −3.23, *p* < 0.001, *r* = 0.40.

Twenty-three of the pre-recorded portions of the trials were repeated a second time because thirteen individuals with schizophrenia and three healthy controls reported failure to notice targets during passive viewing—a statistically significant difference between groups, χ^2^_(1, n = 66)_ = 8.25, *p* = 0.004, phi = −0.35. Twenty-nine individuals with schizophrenia were unable to find at least one of the eight targets (median = 2, range = 0–6) compared to twenty healthy controls (median = 1, range = 0–4); a statistically significant difference as measured by a Wilcoxon SRT, *Z* = −4.21, *p* < 0.001, *r* = −0.52. Healthy controls were more likely to use shortcuts during wayfinding to locate targets than individuals with schizophrenia. Thirty healthy controls were able to find the shortcut for at least one of the trials (median = 3 trials) compared to twenty-two individuals with schizophrenia (median = 1 trial), a statistically significant difference as measured by a Wilcoxon SRT, *Z* = −3.82, *p* < 0.001, *r* = −0.47.

Although patients diagnosed with schizoaffective disorder travelled numerically shorter distances to find targets (median = 15,875, range = 8,490–27,409) than patients diagnosed with schizophrenia (median = 21,492, range = 13,523–51,901) the differences, measured by Mann Whitney U, were not statistically significant, *Z* = −1.53, *p* = 0.13. Similarly, there were no significant differences in the number of targets not found, the number of shortcuts used and the number of prerecorded portions of trials that had to be repeated because of failure to notice targets during passive viewing.

Total target-finding distances across all eight trials were significantly and negatively correlated with RBANS total score (rho = −0.48, *p* < 0.001) and the WAIS III pro-rated full scale IQ (rho = −0.40, *p* < 0.001). Moreover, the differences in standardized z-scores between individuals with schizophrenia and controls in our goal-directed navigation task were as robust as RBANS total scores and almost double that of estimated full scale IQ (Table 2). However, as shown in Table 2, there was some variation across individual indices, with RBANS z-scores for group differences ranging from 0.53 (language) to 0.95 (visualspatial/construction) and WAIS-III performance deficits more than double the effect-size for verbal. These results highlight the importance of assessing visual-spatial abilities in schizophrenia and that our task is at least as sensitive to detecting cognitive deficits as the standardized batteries.

Spearman correlations of distances travelled by individuals with schizophrenia to find targets and clinical symptoms, as measured by SAPS/SANS were non-significant: SAPS (rho =−0.17); SANS (rho =−0.01). Correlations between clinical symptoms and cognitive scores were weak for SAPS (RBANS, *r* = 0.10; WAIS FSIQ, *r* = 0.004) to moderate for SANS (RBANS, *r* = −0.26; WAIS FSIQ, *r* = −0.31). Spearman correlations of distances travelled by individuals with schizophrenia to find targets and antipsychotic medications, computed as chlorpromazine equivalents, were non-significant, rho = −0.06. For the 49 individuals in this study that also completed the Quality of Life scale (25 with schizophrenia, 24 healthy controls), distances travelled across all eight trials correlated negatively with total score results (rho = −0.37, *p* = 0.009); i.e., longer travel distances correlated with lower psychosocial functioning. The correlation between distances travelled and psychosocial function was largely, but not completely, reflective of group differences.

A logistic regression analysis was conducted to predict group membership (schizophrenia or healthy control) using distance travelled, RBANS total score, pro-rated WAIS full scale IQ, and Quality of Life total score as predictors. A test of the full model against a constant only model was statistically significant, indicating that the predictors as a set reliably distinguished between individuals with schizophrenia and healthy controls (chi square = 40.49, *p* < 0.001). Nagelkerke's R^2^ of 0.750 indicated a moderately strong relationship between prediction and grouping. Prediction success overall was 83.7% (84% for schizophrenia, 83.3% for healthy controls). The Wald criterion demonstrated that only Quality of Life (Wald = 8.45) and distance travelled (Wald = 6.67) made significant contribution to prediction (*p* = 0.004, *p* = 0.01, respectively). Neither pro-rated WAIS full scale IQ (Wald = 1.24) nor RBANS (Wald = 2.72) made significant contributions (*p* = 0.23, *p* = 0.1., respectively). Running the model using only Quality of Life and distance travelled as predictors provided an overall prediction success rate of 81.6% (80% for schizophrenia and 83.3% for healthy controls).

## Discussion

The present study investigated the ability of people with schizophrenia to return to a starting position or accurately find a target within a naturalistic virtual cityscape after a single exposure to the target while passively viewing a path taken within the virtual environment. Individuals with schizophrenia had significantly more difficulty than healthy controls matched for age, sex, gaming experience, and education during these rapid, single-trial navigation tasks. Patients travelled significantly further than controls, were less likely to find novel shortcuts to targets, and were more likely to fail in finding the target. Although patients were also more likely to fail to notice the target during passive viewing, the above difficulties persisted even after the passive viewing segments of the trials were repeated to ensure participants saw the relevant target.

Both types of trials posed equal levels of difficulty for individuals with schizophrenia compared to healthy controls and were as effective as RBANS and WAIS III prorated full scale IQ in separating patient from control performance (Table 2). Navigation distance on our task also significantly correlated (negatively) with RBANS total score and the WAIS III pro-rated full scale IQ. We also found that navigation performance across trials was significantly inversely correlated with psychosocial functioning. Logistic regression demonstrated that of the three cognitive variables—distance travelled, RBANS and pro-rated WAIS full scale IQ, only distance travelled made a significant contribution to predicting group membership in a model that included Quality of Life self reports. The combination of Quality of Life self reports and distances travelled demonstrated an overall prediction of group membership success rate of 81.6%. Green and colleagues, in their study of meaningful measures of functioning for use in clinical trials (Green et al., 2011) considered both performance-based and interview-based measures as potential coprimary measures. These results would suggest that a combination of the two might be the more effective strategy.

With respect to clinical symptoms, SANS scores were moderately and SAPS scores weakly correlated with cognitive performance, while correlations between distance travelled and clinical symptoms were non-significant. Schizophrenia symptom correlations with cognitive performance have tended to be inconsistent, but generally minimal for positive symptoms and modest for negative or disorganized symptoms (Gold, 2004; Keefe et al., 2006). The relative independence of cognitive impairment from psychotic symptoms, combined with the presence of cognitive problems before symptom onset (David et al., 1997; Cornblatt et al., 1999; Reichenberg et al., 2002; Niendam et al., 2003; Khandaker et al., 2011; Dickson et al., 2012) and strong relationship to functional outcome (Green, 1996; Harvey et al., 1998; Green et al., 2000, 2004), support the recent focus on cognitive impairment as a unique target for treatment (Hyman and Fenton, 2003).

However, cognitive deficits in schizophrenia are broad and display significant heterogeneity (Joyce and Roiser, 2007). Various factor analytic studies have demonstrated a high level of interrelatedness for domains found to be impaired in schizophrenia (Gladsjo et al., 2004; Dickinson et al., 2006; Keefe et al., 2006; Dickinson and Harvey, 2009). These observations are consistent with the argument that schizophrenia may be a neurodevelopmental disorder (Weinberger, 1987; Seidman, 1990; Lewis and Levitt, 2002) characterized by disturbed functional connectivity (Weinberger et al., 1992; Friston and Frith, 1995; Bullmore et al., 1997; Friston, 1999; Meyer-Lindenberg et al., 2001; Stephan et al., 2006, 2009; Garrity et al., 2007; Liu et al., 2008; Ellison-Wright and Bullmore, 2009; Rotarska-Jagiela et al., 2010). While separate tests for different cognitive domains have shown to be effective for identifying localized dysfunctional areas in the brain, they may not be adequate for understanding functional coordination difficulties. Indeed, the growing use of neuroimaging methodologies such as functional magnetic resonance imaging has led to an increased awareness and emphasis on neural circuits or systems implicated in schizophrenia rather than traditional neuropsychological taxonomies (Minzenberg and Carter, 2012; Gold and Dickinson, 2013).

To the best of our knowledge, all the virtual navigation studies in schizophrenia have used a necessarily constrained paradigm of place learning using rodent models such as the Morris water maze (Morris et al., 1982; Astur et al., 2004; Hanlon et al., 2006, 2012; Folley et al., 2010) and radial arm maze (Olton and Samuelson, 1976; Spieker et al., 2012) or circumscribed environments such as a virtual park (Weniger and Irle, 2008) and virtual town (Ledoux et al., 2013). These were designed to test specific neural structures, principally the hippocampus, and/or specific cognitive domains, such as allocentric memory (Weniger and Irle, 2008; Folley et al., 2010), working and reference memory (Spieker et al., 2012), working and relational memory (Hanlon et al., 2012), and episodic memory (Ledoux et al., 2013). These studies were typically based on a multiple-trial learning paradigm where performance is measured across repetitive trial and error attempts to find one or more game-like rewards, or required extensive exploratory navigation prior to testing to ensure familiarity with all the landmarks subsequently used as targets.

When combined with brain imaging these simplified navigation environments are capable of uncovering dysfunctional circuitry associated with other neural structures and networks in schizophrenia. For example, Astur et al. (2004), in addition to finding reduced hippocampal activation in schizophrenia, also found altered cingulate, insular, and prefrontal cortex activations during virtual navigation. Folley et al. (2010) found anomalous patterns in four widely distributed neural circuits. Hanlon et al. (2012) found lower frontotemporal anatomical connectivity using diffusion tensor imaging. Hanlon et al. (2012) also found that longer path lengths to find the submerged platform in the Morris water maze predicted lower everyday functioning as measured by the UCSD Performance-Based Skills Assessment (UPSA; Patterson et al., 2001). However, the Morris water maze or radial arm maze might not be considered functionally meaningful tests of overall outcome for human clinical trial testing. In addition, Folley et al. (2010), using the virtual Morris water maze, found that impaired performance for both the hidden and exposed platforms was associated with negative symptom severity, suggesting possible motivation difficulties by patients using their paradigm based on a rodent task.

Although not a study of schizophrenia, Spiers and Maguire (2006), using a very realistic model of downtown London (UK) and a single-trial challenge, were able to demonstrate that human goal-directed navigation is not simply a hippocampal place learning phenomenon but rather, a “complex choreography of neural dynamics.” Their model, however, required extensive previous driving experience within this particular city environment.

Our study builds upon these prior findings in presenting a paradigm based on a single-trial attempt at finding typical urban facilities previously seen and located within different areas of a novel realistic virtual city. Each of the four closed-loop trials could be viewed as somewhat similar to trying to find the shortcut to the recreational facility spotted while a passenger during a round trip shopping excursion. Each of the return-path trials were somewhat similar to finding the shortest way back home after being driven to a doctor's appointment. The trial designs of this study have been shown to be as effective as the navigation paradigms used to-date in schizophrenia research in identifying poor functional performance by individuals with schizophrenia and have demonstrated significant correlations with neuropsychological testing and functional outcome. In addition they represent a meaningful test of every day functioning and were judged by most participants as engaging and challenging. The lack of correlation between distance travelled and SANS scores are consistent with these self-reports, and that, unlike the Folley et al. (2010) results based on the virtual Morris water maze, decreased motivation to perform was not apparent during our more naturalistic virtual environment. It should be noted, however, that Hanlon et al. (2012), also using the virtual Morris water maze, found no evidence of amotivation in their results.

The results of these trials support the use of single-trial goal-directed navigation in a naturalistic virtual environment as a measure of cognitive functioning with specific, real life consequences (e.g., getting lost). Future applications will verify successful replication of the task findings, including among subsamples other than the high-functioning patients used here, and more directly explore both specific and global neurocognitive mechanisms accounting for impaired performance among patients. For clinical trial use, the four closed loop trials would appear to be adequate in assessing cognitive functioning, thereby reducing overall trial durations to an average of 15 min plus 5 min for practice and explanation of procedure. In addition, the amenability of this tool in neuroimaging studies in humans and cross referencing results with those of rodent studies will aid in uncovering the aetiology and pathophysiology of schizophrenia.

## Author contributions

Albert H. C. Wong and Todd A. Girard designed the study and wrote the original protocol. John A. Zawadzki and Jason P. Lerch refined the protocol. John A. Zawadzki and Alicia Rodrigues recruited the participants, obtained the informed consents, performed the behavioral testing and collected the data. Ishraq Siddiqui completed various VR software refinements. John A. Zawadzki processed the data and undertook the statistical analysis. Todd A. Girard, George Foussias and Cheryl Grady contributed to data interpretation. John A. Zawadzki wrote the first draft of the manuscript. Albert H. C. Wong supervised the study. All authors discussed the results and contributed to the final version of the paper and have approved its final version.

### Conflict of interest statement

Gary Remington has sat on advisory boards for Roche, Neurocrine, and Synchroneuron, received speaker's fees from Novartis and research support from Neurocrine as well as Medicure. All other authors declare that the research was conducted in the absence of any commercial or financial relationships that could be construed as a potential conflict of interest.
